# Comparison of the Effectiveness of High-Intensity Interval Training in Hypoxia and Normoxia in Healthy Male Volunteers: A Pilot Study

**DOI:** 10.1155/2019/7315714

**Published:** 2019-09-22

**Authors:** Aleksandra Żebrowska, Dariusz Jastrzębski, Ewa Sadowska-Krępa, Marcin Sikora, Camillo Di Giulio

**Affiliations:** ^1^Department of Physiology, Academy of Physical Education in Katowice, Katowice, Poland; ^2^School of Medicine with the Division of Dentistry, Department of Lung Disease and Tuberculosis, Medical University of Silesia, 1 Koziołka St. 41-803 Zabrze, Katowice, Poland; ^3^Department of Neuroscience and Imaging, Dipartimento, University di Madonna delle Piane, Via dei Vestini 31, 66100 Chieti, Italy

## Abstract

**Aims:**

The study investigated the effect of high-intensity interval training in hypoxia and normoxia on serum concentrations of proangiogenic factors, nitric oxide, and inflammatory responses in healthy male volunteers.

**Methods:**

Twelve physically active male subjects completed a high-intensity interval training (HIIT) in normoxia (NorTr) and in normobaric hypoxia (HypTr) (FiO_2_ = 15.2%). The effects of HIIT in hypoxia and normoxia on maximal oxygen uptake, hypoxia-inducible factor-1-alpha, vascular endothelial growth factor, nitric oxide, and cytokines were analyzed.

**Results:**

HIIT in hypoxia significantly increases maximal oxygen uptake (*p*=0.01) levels compared to pretraining levels. Serum hypoxia-inducible factor-1 (*p*=0.01) and nitric oxide levels (*p*=0.05), vascular endothelial growth factor (*p*=0.04), and transforming growth factor-*β* (*p*=0.01) levels were increased in response to exercise test after hypoxic training. There was no effect of training conditions for serum baseline angiogenic factors and cytokines (*p* > 0.05) with higher HIF-1*α* and NO levels after hypoxic training compared to normoxic training (*F* = 9.1; *p* < 0.01 and *F* = 5.7; *p* < 0.05, respectively).

**Conclusions:**

High-intensity interval training in hypoxia seems to induce beneficial adaptations to exercise mediated via a significant increase in the serum concentrations of proangiogenic factors and serum nitric oxide levels compared to the same training regimen in normoxia.

## 1. Introduction

Hypoxic training is commonly used to increase muscle oxidative capacity [1, 2] and exercise performance [3]. The beneficial effects of adaptation to hypoxia have been used for cardiorespiratory control [4, 5], for prevention of metabolic disorders [4, 6], and to induce an improvement in athletic performance and high-altitude acclimatization [7–9]. The mechanisms responsible for these benefits can be grouped into three major categories: adaptation of organs and tissues responsible for oxygen transport [10, 11], improvements in cardiovascular hemodynamics [12–14], and adaptive changes in the immune system [15, 16]. The physiological responses to an acute hypoxic training depend on its various modifications (i.e., natural altitude, simulated altitude, and hypobaric normoxia) [1, 7, 17] and different training regimens [18–20]. Although substantial differences exist between methods of hypoxic training, there is evidence that high-intensity exercise in normobaric hypoxia causes a significantly higher improvement in muscle oxidative capacity with lower inflammatory response compared to other training protocols [7, 9, 19, 21].

Chronic exposure to hypoxia improves oxygen transport by enhancing erythropoietin secretions and the consequential increase in total hemoglobin mass [22, 23], increases cardiorespiratory reserve [5, 11], and improves autonomic nervous system function [4, 11] and skeletal muscle oxidative capacity [10, 24, 25].

The precise molecular mechanism responsible for cardiovascular adaptation in response to hypoxia during exercise training is still not well understood. It has been suggested that training and hypoxia may alter molecular compounds of tissues, such as hypoxia-inducible factor-1*α* (HIF-1*α*) [18, 26], vascular endothelial growth factor (VEGF) [23, 25], and nitric oxide (NO) levels [26–28]. HIF-1*α* targets a number of genes involved in angiogenesis and upregulation of glycolysis and indirectly stimulates the erythropoietin production (EPO) and transcription of the EPO receptor [25]. VEGF is the most important factor affecting angiogenesis by increasing migration and proliferation of endothelial cells [28, 29], and NO is known to play a crucial role in preconditioning and cytoprotection through its vasodilation effect as well as for its ability to modulate mitochondrial function [26, 30, 31]. The upregulation of NO levels, higher inducible NO synthase (iNOS) gene expression, and decrease in the asymmetric dimethylarginine (ADMA) levels have been suggested as the beneficial endothelial-dependent vasodilation mechanisms in response to hypoxia [31–34].

The exposure to hypoxia can also stimulate the release of several transcriptional factors, which play a central role in stimulating the proinflammatory cytokines such as interleukin-6 (IL-6) and tumor necrosis factor-alpha (TNF-*α*) [15, 35, 36]. TNF-*α* is a proinflammatory cytokine and its concentration also rises in acute inflammation state such as muscle microdamages. Therefore, a decrease in its level in response to a high-intensity exercise could indicate an adaptive reduction in the inflammatory response to metabolic stress induced by exercise. Hence, a better understanding of molecular and physiological responses to hypoxic training will benefit athletes and coaches of sports discipline and events held in high-altitude conditions. It has been evidenced that high-intensity hypoxic training may induce beneficial hematological and cardiorespiratory changes mediated via a significant increase in the serum concentrations of proangiogenic factors and serum nitric oxide levels compared to the same training regimen in normoxia [12, 18, 37]. Therefore, this study aimed at evaluating the comparison of the effectiveness of interval training in normobaric hypoxia and normoxia on serum concentrations of proangiogenic factors, nitric oxide, cytokine responses, and cardiorespiratory function in healthy male volunteers. It was hypothesized that markers NO and HIF-1-alpha might be more sensitive than standard angiogenic biomarkers in the early detection of hypoxia-induced cardiorespiratory adaptation in endurance-trained athletes.

## 2. Materials and Methods

### 2.1. Subjects

Study members were recruited from a group of physically active male volunteers (aged 24.4 ± 4.0 years), extramural students of the Academy of Physical Education, who gave written, informed consent to take part in the study. Before the study, all the participants were assessed for body composition using a model In Body220 (Biospace Inc., Seoul, Korea) analyzer and completed a survey on their training history. After baseline examination, participants were randomly assigned, with a 1 : 1 assignment, to either the hypoxia training group (HypTr) or the normoxia training group (NorTr) (utilizing computer-generated random numbers): 6 subjects were assigned to the HypTr group and 6 subjects were assigned to the NorTr group. Both groups completed a 3-week training program in normoxia (Nor) and a 3-week training program in hypoxia (Hyp). Participants of each group were crossed over to the opposite training protocol after a 6-week break. The exercise and training programs were conducted in the laboratory. During the study, all participants monitored their training and recreational activities, i.e., jogging, running, cycling, and mountain tracking. The men training volume per week was 240 ± 25 minutes of moderate exercise and 120 ± 30 minutes high-intensive exercise. The training was not significantly different between the groups. All of the participants were born and living at sea level, and their training status, expressed as maximal oxygen consumption (VO_2max_), was 54.2 ± 2.6 ml/kg/min (Table 1). No significant difference between the two groups was observed in the training status outside the hypoxic chamber (The Altitude Trainer Hypoxico System (HYP-123 Hypoxic Generator, Lowoxygen Technology GmbH, Berlin, Germany)) and during the washout period. The exclusion criteria used in order to eliminate factors which might influence the vascular parameters were as follows: evidence of hemodynamic dysfunction, inflammatory diseases in the preceding 3 months, and cigarette smoking within 12 h before the examination.

The study was approved by the local Bioethical Research Committee (Ethics Committee decision KBN3/2016) and conducted in accordance with the Declaration of Helsinki of the World Medical Association.

### 2.2. Exercise Protocol

The exercise protocol was divided into four visits to the laboratory. During the first visit before (preTr) both training protocols (HypTr vs. NorTr), all participants were subjected to an incremental exercise test under normoxia and hypoxia. At baseline, the tests were used to measure the individual aerobic performance (maximal oxygen uptake, VO_2max_) and lactate threshold (LAT) to calculate the total sample size and to determine the intensity of interval training protocols in hypoxic and normoxic conditions. The incremental exercise tests were repeated after the 3-week training program (postTr) for the normoxia and hypoxia groups. After the 6-week washout period, all participants performed the first testing session (preTr), and the last incremental exercise tests were performed after the 3-week training program for the NorTr and HypTr groups.

The test started with a 3-minute warm-up; the intensity was then increased by 30 W every 3 minutes up to maximal exercise intensity. Heart rate (HR) was continuously monitored (PE-3000 Sport-Tester, Polar Inc., Finland). Blood pressure (SDB/DBP) was measured in duplicate with a sphygmomanometer (HEM-907 XL, Omron Corporation, Japan) before and immediately after exercise. Pulmonary ventilation (VE), oxygen uptake (VO_2_), and carbon dioxide output (CO_2_) were measured continuously from the 6th min prior to the exercise test and throughout each stage of the exercise test using the Ergospirometry Metalyzer 3B-2R (Cortex, Germany). Criteria for termination of VO_2max_ were voluntary exhaustion, respiratory ratio equal to or exceeding 1.15 (RER ≥1.1), a VO_2_ plateau, and blood lactate concentration ≥8.0 mmol/L.

### 2.3. High-Intensity Interval Training (HIIT)

All subjects were divided randomly into HIIT in normoxia (NorTr, FIO_2_ = 20.9%, *p* = 990 hPa) and hypoxia (HypTr FIO_2_ = 15.2% *p* = 990 hPa). After 15-minute rest in sitting position for acclimatization to hypoxia/normoxia conditions, a warm-up consisting of 5-minute cycling at intensity 30 Watts was performed. HIIT sessions consist of an interval cycling exercise with an intensity of 120% of subjects individual lactate threshold (LAT) calculated individually for each athlete when exposed to normoxic and hypoxic conditions. Each training session consisted of 6 bouts of exercise with a duration of 5 minutes, intermittent with 5-minute rest after each bout of exercise. The training sessions were performed three times per week during three weeks of each training protocol (NorTr vs. HypTr) with the 6-week detraining period to wash out the training effects. All training sessions were conducted in an environmentally controlled chamber using an electromagnetically braked cycle ergometer (Lode B.V., Groningen, the Netherlands). The Altitude Trainer Hypoxico System (HYP-123 Hypoxic Generator, Lowoxygen Technology GmbH, Berlin, Germany) was used in the hypoxia training protocol.

A few days before the examination, the subjects were asked to abstain from exercise and were put on their standardized normocaloric diet. The protocol was based on the laboratory studies (ambient conditions: 21°C, 60% relative humidity) after an overnight fast.

### 2.4. Biochemical Analyses

Physiological variables and biochemical variables were measured after 15-minute rest (rest) and immediately after maximal exercise test (max) in hypoxia and normoxia. At the beginning and at the end of each study protocol (HypTr and NorTr), all subjects reported to the laboratory and had venous blood drawn for the determination of hypoxia-inducible factor-1-alpha (HIF-1*α*), nitric oxide (NO), asymmetric dimethylarginine (ADMA), vascular endothelial growth factor (VEGF), transforming growth factor-beta (TGF-*β*), tumor necrosis factor-alpha (TNF-*α*), and interleukin-6 (IL-6) and interleukin-1-beta (IL-1*β*) concentrations.

The levels of HIF-1*α* were measured by enzyme-linked immunosorbent assay (ELISA) kit (BlueGene Biotech, China). The intra-assay coefficient of variation was <4.4%, and the interassay coefficient of variation was <5.6% and sensitivity was 0.5 pg/mL. The measurements of total NO and nitrite/nitrate and ADMA were performed using enzyme-linked immunosorbent assay (ELISA—R&D Systems Inc., Minneapolis, USA). The sensitivity of total NO/nitrite/nitrate assay was 0.25 *μ*mol/l. Intra-assay and interassay coefficients of variation for total NO/nitrite/nitrate were <2.5% and <4.6%, respectively. ADMA test disclosed values as low as 0.05 *μ*mol/l. The intra-assay coefficient of variation was <9.8%, and the interassay coefficient of variation was <7.5%.

The levels of VEGF and TGF-*β* were measured by enzyme-linked immunosorbent assay (ELISA) kit (BlueGene Biotech, China). Intra-assay and interassay coefficients of variation for VEGF and TGF-*β* were <4.4% and <5.6%, respectively. Serum TNF-*α* levels were measured by DIAsource ImmunoAssays, Belgium. The intra-assay coefficient of variation was <4.6%, and the interassay coefficient of variation was <5.6%. The sensitivity of VEGF, TGF-*β*, and TNF-*α* assays was 1.0 pg/mL, 0.7 pg/mL, and 1.0 pg/mL, respectively.

Serum levels of IL-6 were measured by using Human IL-6 High Sensitive ELISA kit (Diaclone, France), and IL-1*β* levels were measured by using Human IL-1*β* ELISA kits (Diaclone, France). Intra-assay and interassay coefficients of variation for of IL-6 were <4.4% and <6.4%, respectively, with a sensitivity of 0.7 pg/mL. Intra-assay and interassay coefficients of variation for of IL-1*β* were <5.1% and <8.6%, respectively, with a sensitivity of 0.3 pg/mL.

Red blood cells (RBC), white blood cells (WBC), lymphocytes (LYM), monocytes (MON), absolute neutrophil counts (ANC), and haemoglobin (HGB) levels were measured (ABX MICROS 60, HORIBA). Blood lactate concentrations (LA) were determined using Biosen C-line method (EKF Diagnostic GmbH); blood gases and acid-base balance were also analyzed (Rapidlab 348; Bayer Diagnostics, Germany). To avoid the effects of diurnal variations, blood samples were taken in the morning at the same period (between 8.00 and 9.00 am). The degree of haemoconcentration (%) was calculated according to the formula of subtracting the peak haematocrit with the minimum haematocrit recorded and multiplying by 100; all biochemical variables levels were corrected according to plasma volume.

### 2.5. Statistical Analysis

Analyses were performed using commercially available software (the Statistica package v. 12; StatSoft Poland, 12.0). All data given from the statistical methods were presented as the mean and standard deviation (SD). The total sample size was calculated using the Altman nomogram and alpha value of 0.05 for 0.07 test power. Shapiro–Wilk, Levene's, and Mauchly's tests were used in order to verify the normality, homogeneity, and sphericity of the sample's data variances, respectively. Verifications of the differences between analyzed variables (pretraining vs. posttraining) and groups (normoxia vs. hypoxia) were carried out using two-way ANOVA with correction for repeated measurements. The significance of the differences between the groups was verified with the Bonferroni post hoc test. Correlation coefficients between all the variables were determined with the Spearman test. For all procedures, a level of *p* < 0.05 was selected to indicate statistical significance.

## 3. Results

The analyses of pulmonary and cardiovascular variables before and after completion of the HIIT in hypoxia (HypTr) and normoxia (NorTr) are presented in Table 2. Prior to the start of both training sessions, no significant differences regarding VO_2max_ were found. A repeated-measures two-way ANOVA revealed the significance of training and group interaction effects on relative VO_2max_ (*F* = 15.5; *p* < 0.001) and maximal power levels (*P*_max_) (*F* = 12.8; *p* < 0.01). After the HIIT in hypoxia, athletes were characterized by significantly higher maximum oxygen uptake (*p*=0.01) compared to pretraining levels (Table 2). No significant effects of training-group interaction were observed in serum lactate concentrations (LA), pulmonary ventilation (VE), and as to cardiovascular variables (Table 2). There was a significant increase in VE levels after HypTr and NorTr compared to pretraining values (*p*=0.05). Significant differences were found in oxygen arterial saturation in response to the training (*F* = 34.6; *p* < 0.001) and oxygen conditions (*F* = 90.8; *p* < 0.001). A significant positive correlation was observed between VO_2max_ and VE_max_ (*p*=0.001; *r* = 0.81).

The results of hematological variables and blood cells count are presented in Table 3. Our results revealed significant effects of HypTr on ANC (*F* = 7.7 *p* < 0.05). A significantly higher ANC was observed after HypTr compared to pretraining values (*p*=0.04). ANOVA revealed significant effects of HypTr on WBC count (*F* = 14.2 *p* < 0.01) and higher values in response to HypTr (*p*=0.04). High-intensity interval training in hypoxia had significant effects on hemoglobin concentrations (*F* = 8.1 *p* < 0.05) and haematocrit (*F* = 7.2 *p* < 0.01) with no significant effect on RBC and percent of reticulocytes (Table 3).

**Figure 1:**
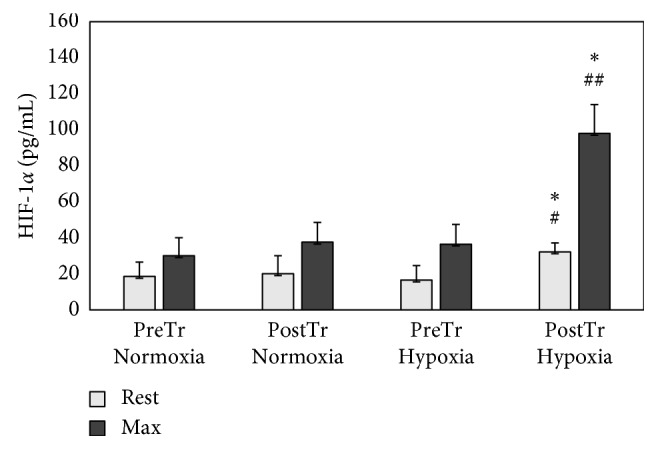
Hypoxia-inducible factor-1-alpha (HIF-1*α*) pretraining (preTr) and posttraining (postTr) levels at rest and at maximal exercise test (max) in normoxia and hypoxia. Data are means and SD. ^*∗*^*p* < 0.01, significant difference between preTr and postTr; ^#^*p* < 0.05, ^##^*p* < 0.01, significant difference between normoxia and hypoxia.

**Figure 2:**
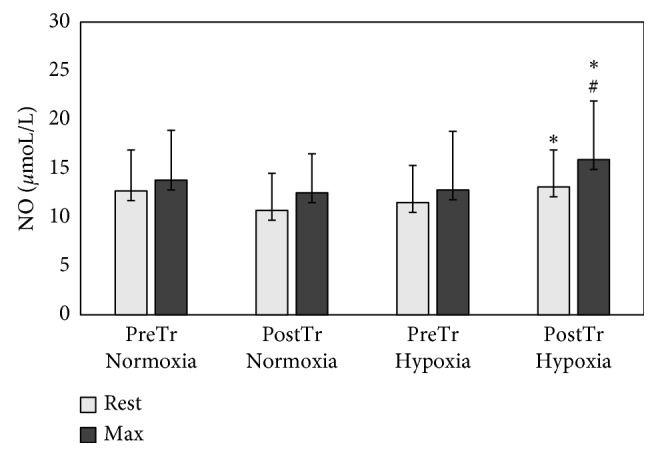
Nitric oxide (NO) pretraining (preTr) and posttraining (postTr) levels at rest and at maximal exercise test (max) in normoxia and hypoxia. Data are means and SD. ^*∗*^*p* < 0.01, significant difference between preTr and postTr; ^#^*p* < 0.05, significant difference between normoxia and hypoxia.

ANOVA revealed a significant effect of the hypoxia on the baseline (*F* = 9.1 *p* < 0.01) and postexercise (*F* = 7.7 *p* < 0.05) serum HIF-1*α* levels. Significantly higher pre- and postexercise HIF-1*α* levels were observed in response to HypTr compared to pretraining levels (*p*=0.02 and *p*=0.01, respectively) (Figure 1). Hypoxia training increased baseline (*p*=0.01) and postexercise NO levels (*p*=0.05) (Figure 2). The significance of training and group interaction effects on NO (*F* = 5.7; *p* < 0.01) was observed. No significant differences between the oxygen conditions and training were observed as to ADMA levels (*p* > 0.05). ANOVA revealed a significant effect of training on VEGF levels (*F* = 8.5; *p* < 0.01). Higher postexercise levels of VEGF in response to HypTr (*p*=0.04) and NorTr (*p*=0.03) compared to pretraining levels were observed (Table 4). HIIT in normobaric hypoxia resulted in a significant increase in serum baseline (*p*=0.02) and postexercise (*p*=0.01) TGF-*β* levels compared to pretraining values with no significant effects on TNF-*α* levels. ANOVA revealed significant effects of training-group interaction on IL-1*β* levels (*F* = 10.6; *p* < 0.01). After the HIIT in hypoxia, significantly higher postexercise IL-1*β* concentrations were found compared to the pretraining level (*p*=0.01). TGF-*β*_max_ was found to be correlated with total maximal oxygen uptake (*p*=0.001; *r* = 0.73).

## 4. Discussion

Previous studies have demonstrated that normobaric hypoxia combined with endurance training is the most sufficient method for improving maximum oxygen uptake [7, 12, 38] and exercise performance [39, 40]. This physiological adaptation contributes to enhanced aerobic capacity via an increase in erythropoietic effects [41] and peripheral factors to oxygen transport and utilization [42]. However, the effect of hypoxic training on markers of angiogenesis has received little attention. Therefore, this study investigated the effect of 3 weeks of HIIT in hypoxic and normoxic conditions on serum concentrations of proangiogenic factors, nitric oxide, and inflammatory responses in recreationally endurance-trained participants. The major findings of this study show that (i) HIIT in hypoxia is effective for improving aerobic performance and maximum oxygen uptake; (ii) HIIT induces higher concentration of serum proangiogenic factors compared to the same training regimen in normoxia; (iii) HIIT in hypoxia leads to improvement of endothelial-dependent vasodilation mechanisms due to stimulation of NO levels.

### 4.1. Improvements of Aerobic Performance by Hypoxic Training

Given that a combination of HIIT and hypoxia may lead to improvement of aerobic endurance [38, 39], our study showed a significant increase in VO_2max_ after three weeks of training while inspiring 15% O_2_ three times a week compared to pretraining values. Hypoxia combined with training did not influence RBC count or RET%; however, higher HCT and Hb levels have been observed compared to pretraining values. Interestingly, a marginal (1.1%) increase in HCT and Hb (2.6%) in hypoxia compared with normoxia training group was observed. Our data are in accordance with previous studies, which reported that the exposure to hypoxia during 3-week period (1-2 h per day) may be insufficient to modify hematological variables [19, 38, 40], but hypoxic training method can contribute to the improvement of nonhematological adaptive mechanisms [17, 29, 30]. It is possible that in our study, an increase in HCT and Hb can be related to the effect of haemoconstriction (1.2%) rather than due to the acceleration of erythropoiesis. In this study, we are not able to precisely define whether the exposure to hypoxia stimulates hematological changes due to higher erythropoietin (EPO) concentrations [40, 43, 44]; thus, this should be the focus of future studies.

The increase in aerobic capacity following 3 weeks of hypoxic training may also contribute to an increase in respiratory performance and cardiac function. Also, these differences in maximal oxygen uptake can be related to different experimental settings in the hypoxic chamber and differences in subjects recreational exercise activity. In the present study, HIIT in hypoxia has little influence on respiratory performance and cardiac function. It cannot be excluded, however, that hypoxia might have a beneficial vasodilator effect on pulmonary vasculature and oxygenation.

### 4.2. Angiogenic Factors in response to Different HIIT and Hypoxia

The present study demonstrates that the combination of hypoxia with interval training increased baseline and postexercise HIF-1*α* and NO levels compared to training in normoxia. Consequent changes in postexercise proangiogenic biomarkers and endothelium-dependent vasodilator function in response to 3 weeks of HIIT in hypoxia might, in turn, have significantly increased athletes' exercise performance compared to the same training program performed under normoxic conditions.

The physiological role of HIF-1*α* response to hypoxic training is not well understood, and it seems that it might modulate oxygen-sensitive central and peripheral mechanisms affecting the adaptation processes of respiratory [33, 45, 46] and cardiovascular systems [23, 47–50]. The impaired oxygen homeostasis seems to involve the carotid body and its role to modulate ventilation and tissue oxygenation. The human carotid body expresses HIF-1*α* which induces upregulation of nitric oxide synthase (NOS-1) and vascular endothelial growth factor (VEGF), and the levels of expressed proteins are greater in hypoxic than in normoxic conditions [51]. In the animal study, the exposure to hypoxia with HIIT 3 days per week for 4 weeks with an intensity of 100% VO_2max_ was associated with higher HIF-1*α* level and VEGF mRNA expression compared to normoxic training [52]. It has also been reported that hypoxia after resistance training significantly increases plasma VEGF concentrations [24]. These results suggest that all the aforementioned changes may also lead to increased muscle endurance capacity and the promotion of angiogenesis in skeletal muscles. However, we can find in the literature reverse data pointing to a significant effect of normobaric hypoxia training on muscle oxygenation during intermittent sprinting without significantly greater expression of muscle HIF-1*α* in humans [25, 53].

In our study, high-intensity training resulted in a significant increase in serum postexercise VEGF levels; however, no significant effects of hypoxia were observed. In agreement with our study, no significant changes in the VEGF and VEGF mRNA expression were observed after low-intensity training regimen under hypoxic conditions, even though an increase in the concentrations of HIF-1*α* mRNA was detected [12]. Previously, in a human study, 5-week training of 45-minute exercise bouts with blood flow restriction significantly influenced VEGF-A systems measured in biopsies from the vastus lateralis muscle [54]. It should be emphasized that this research did not account for serum angiogenesis factors, although there was a suggested relationship between higher VEGF-A mRNA expression and neovascularization processes in skeletal muscle [52, 54].

Considering that maximal oxygen uptake is correlated with changes in vascular hemodynamics, we hypothesized that the stimulation of the NO vasodilator system might be an important marker of the effectiveness of hypoxic training [33, 55, 56]. This study has found significant differences in the baseline and postexercise serum NO levels in response to HIIT in hypoxia compared to normoxia. The aforementioned effects were associated with no significant differences in ADMA between HypTr and NorTr. Thus, our results suggest that HIIT and hypoxia by combining the effects of vasoconstriction and repeated exposure to shear stress due to increased blood viscosity upregulation of endothelial NO synthase gene expression enhance vascular NO production and its vasodilation effects [57–59]. Chronic hypoxia and exercise training affect the NO content and may be involved in its delayed protective effects [32, 56]. As upregulation of endothelial NOS activity in response to training in hypoxic might enhance endothelial NO production, these higher NO levels in response to HIIT in hypoxia may correlate positively with oxygen consumption [60].

Hypoxia has also been found to induce an inflammatory response, mediated by leukocytes and manifested by elevated concentrations of the proinflammatory cytokines [15, 16, 35, 36]; however, there are conflicting data that these proteins may contribute to hypoxia-related neovascularization processes in healthy individuals [57, 61]. In the present study, hypoxia and training activated inflammatory cells (neutrophils and macrophages) and stimulated higher serum TGF-*β* and IL-1*β* levels. TGF-*β* stimulates angiogenesis by inducing apoptosis of endothelial cells, which is vital for normal vascular structure. The performance of the cardiovascular system might be affected by a greater stimulation of the interleukin (IL-6) and tumor necrosis factor (TNF *α*) in endothelial cells [61, 62]. It has also been stated that IL-6 mRNA expression significantly decreased in response to hypoxic training, indicating that hypoxic training results in a greater generation of NO than normoxic training. In our study, similar effects of HypTr and NorTr were observed in relation to IL-6 and TNF-*α* levels. The lack of significant TNF-*α* and IL-6 increases in response to a high-intensity interval training could indicate an adaptive reduction in the inflammatory response to metabolic stress induced by exercise in hypoxia [44, 63].

## 5. Limitations

There are several limitations but also some favorable aspects to our study. First, we could not investigate long-term hematological changes due to erythropoietic effects and the influence of higher EPO production on proinflammatory cytokine kinetics. Second, we could not use an ultrasound evaluation of the arterial structure and function. However, the cardiovascular adaptation to both training regimen determined by investigating the basal angiogenic factors and protocol characteristics allowed objective analyses and important conclusions. The sample size was limited, which was due to excluding from analysis the participants who did not complete all study protocols. Whether or not this high-intensity hypoxia training regimen will result in long-term benefits needs further investigation.

## 6. Conclusions

High-intensity interval training with hypoxia is sufficient to induce physiological adaptations mediated via a significant increase in the serum hypoxia-inducible factor-1*α*, vascular growth factor, and serum nitric oxide levels. Two potential mechanisms may be taken into consideration: the first is the induction of the endothelial system and the second is the improvement of vasodilation conditions. Considering that maximal oxygen uptake is correlated with changes in vascular hemodynamics, the stimulation of the angiogenic factors and the nitric oxide vasodilator system during high-intensity interval training might be the important factors that contribute to the effectiveness of hypoxic training.

## Figures and Tables

**Table 1:** Subject's anthropometric characteristics.

Variable	*n* = 12
Age (yr)	24.4 (4.0)
Body height (cm)	180.6 (5.9)
Body weight (kg)	72.2 (4.4)
BMI (kg/m^2^)	21.8 (1.2)
HR_rest_ (1/min)	54.2 (2.6)
SBP_rest_ (mm Hg)	120.0 (15.0)
DBP_pre_ (mm Hg)	77.0 (5.0)
VO_2max_ (mL/kg/min)	54.2 (2.6)

Data are means and SD.

**Table 2:** Maximal oxygen uptake (VO_2max_) and physiological results before and after training in both training groups.

Variables	Pretraining		Posttraining		*p*	95% confidence interval for differences		
	Mean	SD	Mean	SD		Mean differences	Lower bound	Upper bound
*Hypoxia group*								
VO_2max_ (mL/kg/min)	53.2	2.6	59.4	4.6	0.01	6.2	−8.9	−2.7
LA_max_ (mmol/L)	11.1	1.8	10.4	1.5	0.06	−0.7	0.1	1.3
VE_max_ (L/min)	163.2	9.6	166.9	13.8	0.05	3.7	−12.2	−2.2
HR_max_ (1/min)	189.0	8.0	191.0	4.0	0.07	2.0	0.1	2.8
SBP_max_ (mm Hg)	160.0	12.0	162.0	13.0	0.11	2.0	−14.1	−4.1
DBP_max_ (mm Hg)	77.0	8.0	72.0	6.0	0.06	−5.0	−11.2	−2.4
SatO_2max_ (%)	94.8	3.6	91.5	2.4	0.01	−3.3	0.8	4.4
pO_2max_ (mmHg)	88.3	1.7	68.2	1.2	0.01	−20.1	17.3	22.7
pCO_2max_ (mmHg)	34.1	1.7	33.4	1.2	0.05	−0.7	−0.4	1.2
*P*_max_ (Watt)	277.3	16.1	293.6	15.7	0.05	16.4	−30.2	−2.5

*Normoxia group*								
VO_2max_ (mL/kg/min)	54.2	2.6	55.4	4.8	0.47	1.2	−2.9	1.4
LA_max_ (mmol/L)	11.2	1.8	10.6	1.6	0.33	−0.6	−0.3	0.9
VE_max_ (L/min)	160.4	12.5	165.4	13.0	0.05	5.0	−7.8	−1.5
HR_max_ (1/min)	190.0	6.0	191.0	3.0	0.06	1.0	−4.0	0.1
SBP_max_ (mm Hg)	156.0	10.0	155.0	9.0	1.00	−1.0	−8.9	−4.1
DBP_max_ (mm Hg)	77.0	8.0	76.0	8.0	0.91	−1.0	−4.8	−3.7
SatO_2max_ (%)	98.8	3.6	96.8	4.1	0.08	−2.0	1.8	3.1
pO_2max_ (mmHg)	88.3	1.7	87.8	1.8	0.17	−0.5	−0.2	1.4
pCO_2max_ (mmHg)	35.1	1.7	34.2	1.3	0.08	−0.9	−0.4	1.2
*P*_max_ (Watt)	280.1	15.1	290.0	9.2	0.06	18.9	−31.6	−4.8

**Table 3:** Hematological results before and after training in both training groups.

Variables	Pretraining		Posttraining		*p*	95% confidence interval for differences		
	Mean	SD	Mean	SD		Mean differences	Lower bound	Upper bound
*Hypoxia group*								
LYM (10^9^/L)	2.1	0.9	2.2	0.5	0.28	0.1	−0.5	−0.1
ANC (10^9^/L)	2.5	0.7	3.2	1.2	0.04	0.7	−1.4	0.6
MON (10^9^/L)	0.6	0.3	0.5	0.3	0.25	−0.1	−0.1	0.2
WBC (10^9^/L)	5.2	1.7	5.8	1.1	0.04	0.6	−1.4	0.4
HGB (g/L)	15.4	2.1	15.8	0.6	0.02	0.4	−0.6	−0.2
HCT (%)	44.8	1.8	45.3	2.1	0.04	0.5	−0.9	0.1
RBC (10^12^/L)	5.0	0.8	5.2	0.4	0.06	0.2	−0.4	0
RET (%)	1.0	0.2	1.2	0.2	0.05	0.2	−0.2	−0.1

*Normoxia group*								
LYM (10^9^/L)	2.1	0.6	2.1	0.7	0.69	0	−0.1	0.1
ANC (10^9^/L)	2.4	0.8	2.1	1.6	0.14	−0.3	−0.1	0.6
MON (10^9^/L)	0.6	0.2	0.7	0.3	0.05	0.1	−0.2	0
WBC (10^9^/L)	5.1	1.9	5.2	2.0	0.66	0.1	−0.8	0.5
HGB (g/L)	15.3	1.0	15.1	0.9	0.16	−0.2	−0.1	0.3
HCT (%)	44.2	2.0	44.0	2.8	0.94	0.2	−0.8	0.8
RBC (10^12^/L)	5.1	0.4	5.0	0.2	0.38	−0.1	−0.1	0.2
RET (%)	1.1	0.1	1.0	0.1	1.00	−0.1	0	0

**Table 4:** Angiogenic factors and cytokine results before and after training in both training groups.

Variables	Pretraining		Posttraining		*p*	95% confidence interval for differences		
	Mean	SD	Mean	SD		Mean differences	Lower bound	Upper bound
*Hypoxia group*								
HIF-1*α*_rest_ (ng/mL)	16.6	7.8	32.2	5.2	0.02	15.6	−20.2	−9.6
HIF-1*α*_max_ (ng/mL)	36.5	11.2	98.0	16.4	0.01	61.5	−74.7	−47.3
NO_rest_ (*μ*moL/L)	11.5	3.8	13.1	3.8	0.01	1.6	−2.4	−0.4
NO_max_(*μ*moL/L)	12.8	6.1	15.9	6.0	0.05	3.1	−7.3	0.5
ADMA_rest_ (*μ*moL/L)	0.4	0.2	0.6	0.1	0.06	0.2	−0.3	−0.02
ADMA_max_ (*μ*moL/L)	0.7	0.3	0.6	0.3	0.07	−0.1	0	0.4
VEGF_rest_ (pg/mL)	15.9	8.4	20.6	7.5	0.10	4.7	−9.9	1.0
VEGF_max_ (pg/mL)	9.5	6.7	17.6	8.1	0.04	8.1	−16.1	−0.4
TGF-*β*_rest_ (pg/mL)	118.5	10.8	134.6	19.4	0.02	16.1	−23.0	−9.2
TGF-*β*_max_ (pg/mL)	129.4	22.6	158.0	34.8	0.01	28.6	−48.4	−8.6
TNF-*α*_rest_ (pg/mL)	24.2	9.8	35.7	14.2	0.07	11.5	−16.8	−5.3
TNF-*α*_max_ (pg/mL)	36.5	11.0	42.3	18.1	0.12	5.8	−13.8	1.8
IL-6_rest_ (pg/mL)	1.3	0.6	1.9	1.5	0.06	0.6	−1.3	−0.2
IL-6_max_ (pg/mL)	1.4	0.7	2.1	1.3	0.06	−0.1	−1.1	−0.1
IL-1*β*_rest_ (pg/mL)	3.1	1.9	5.9	3.6	0.06	2.8	−4.2	−2.0
IL-1*β*_max_ (pg/mL)	3.8	2.0	8.1	3.6	0.01	4.3	−0.3	0.2

*Normoxia group*								
HIF-1*α*_rest_ (ng/mL)	18.6	8.1	20.1	10.7	0.61	1.5	−8.0	4.9
HIF-1*α*_max_ (ng/mL)	30.1	10.4	37.6	11.1	0.05	7.5	−10.8	−2.4
NO_rest_ (*μ*moL/L)	12.7	4.2	10.7	3.8	0.06	−2.0	1.0	3.1
NO_max_(*μ*moL/L)	13.8	5.1	12.5	4.0	0.90	−1.3	−0.4	4.2
ADMA_rest_ (*μ*moL/L)	0.5	0.3	0.5	0.2	0.34	0	−0.1	0.0
ADMA_max_ (*μ*moL/L)	0.6	0.4	0.7	0.2	0.85	0.1	−0.2	0.2
VEGF_rest_ (pg/mL)	17.2	14.1	22.6	15.8	0.27	5.4	−13.1	4.1
VEGF_max_ (pg/mL)	8.4	7.3	17.4	11.3	0.03	9.0	−18.6	−0.9
TGF-*β*_rest_ (pg/mL)	121.5	18.8	131.0	18.8	0.18	9.5	−19.9	4.3
TGF-*β*_max_ (pg/mL)	127.8	27.0	143.0	42.0	0.04	15.2	−26.6	14.8
TNF-*α*_rest_ (pg/mL)	24.2	9.8	35.1	10.4	0.06	10.9	−16.1	−5.1
TNF-*α*_max_ (pg/mL)	34.8	10.3	34.3	7.7	0.91	−0.5	−6.8	7.6
IL-6_rest_ (pg/mL)	1.2	0.8	0.9	0.6	0.22	−0.3	−0.2	0.8
IL-6_max_ (pg/mL)	1.3	0.9	1.1	1.0	0.21	−0.2	−0.1	0.6
IL-1*β*_rest_ (pg/mL)	3.3	2.0	3.1	3.3	0.72	−0.2	−0.6	0.9
IL-1*β*_max_ (pg/mL)	3.5	2.6	3.3	1.7	0.03	−0.2	−0.5–2	0.4

## Data Availability

The data used to support the findings of this study are available from the corresponding author upon request.
